# Regulation of the *F11, Klkb1, Cyp4v3* Gene Cluster in Livers of Metabolically Challenged Mice

**DOI:** 10.1371/journal.pone.0074637

**Published:** 2013-09-16

**Authors:** Huma Safdar, Audrey C. A. Cleuren, Ka Lei Cheung, Frank J. Gonzalez, Hans L. Vos, Yusuke Inoue, Pieter H. Reitsma, Bart J. M. van Vlijmen

**Affiliations:** 1 Einthoven Laboratory for Experimental Vascular Medicine, Leiden University Medical Center, Leiden, The Netherlands; 2 Department of Thrombosis and Hemostasis, Leiden University Medical Center, Leiden, The Netherlands; 3 Laboratory of Metabolism, National Cancer Institute, Bethesda, Maryland, United States of America; 4 Department of Chemistry and Chemical Biology, Graduate School of Engineering, Gunma University, Kiryu, Gunma, Japan; INRA, France

## Abstract

Single nucleotide polymorphisms (SNPs) in a 4q35.2 locus that harbors the coagulation factor XI (*F11*), prekallikrein (*KLKB1*), and a cytochrome P450 family member (*CYP4V2*) genes are associated with deep venous thrombosis (DVT). These SNPs exert their effect on DVT by modifying the circulating levels of FXI. However, SNPs associated with DVT were not necessarily all in *F11*, but also in *KLKB1* and *CYP4V2*. Here, we searched for evidence for common regulatory elements within the 4q35.2 locus, outside the *F11* gene, that might control FXI plasma levels and/or DVT risk. To this end, we investigated the regulation of the orthologous mouse gene cluster under several metabolic conditions that impact mouse hepatic *F11* transcription. In livers of mice in which HNF4α, a key transcription factor controlling *F11*, was ablated, or reduced by siRNA, a strong decrease in hepatic *F11* transcript levels was observed that correlated with *Cyp4v3* (mouse orthologue of *CYP4V2*), but not by *Klkb1* levels. Estrogens induced hepatic *F11* and *Cyp4v3*, but not *Klkb1* transcript levels, whereas thyroid hormone strongly induced hepatic *F11* transcript levels, and reduced *Cyp4v3*, leaving *Klkb1* levels unaffected. Mice fed a high-fat diet also had elevated *F11* transcription, markedly paralleled by an induction of *Klkb1 and Cyp4v3* expression. We conclude that within the mouse *F11*, *Klkb1*, *Cyp4v3* gene cluster, *F11* and *Cyp4v3* frequently display striking parallel transcriptional responses suggesting the presence of shared regulatory elements.

## Introduction

Blood coagulation serine protease factor XI (FXI) contributes to hemostasis by activating coagulation factor IX [1]. Although bleeding associated with FXI deficiency is relatively mild, there has been a resurgence of interest in FXI, because candidate gene studies revealed a role of high FXI levels as a risk factor for venous thrombosis [2,3]. Factor XI, like the blood coagulation protease factors II, VII, IX, X, XII and XIIIb, is produced primarily in hepatocytes. FXI is a dimeric serine protease that is structurally closely related to prekallikrein, a serine protease that is also involved in the intrinsic blood coagulation pathway [4]. Both factor XI and prekallikrein are activated by active coagulation factor XII [5-7].

**Figure 1:**
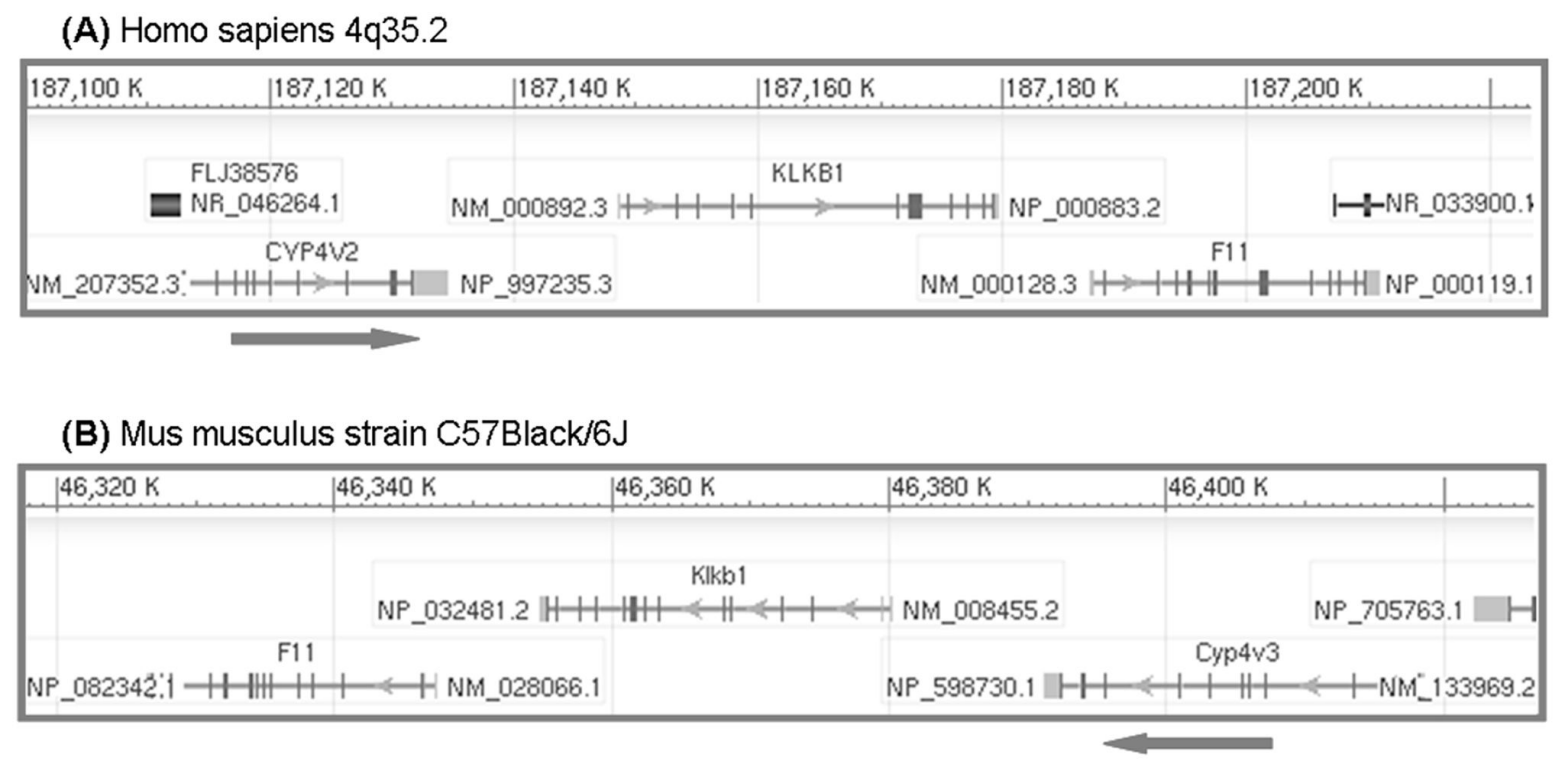
F11 gene locus organization in human (A) and mice (B). Vertical bars representing exons, and lines representing introns. Arrows show the orientation of transcription. Source; http://www.ncbi.nlm.nih.gov/.

The genes encoding FXI (*F11*) and prekallikrein (*KLKB1*) are located in tandem on the long arm of chromosome 4 (4q35.2) directly downstream from the cytochrome P450 family member (*CYP4V2*) gene and family with sequence similarity 149 A (*FAM149A*, Figure 1). The size of this 4q35.2 locus is approximately 200 kb (197.715 bp; position 187297059–187494774). It is assumed that CYP4V2, which has a role in fatty acid metabolism [8], is not involved in coagulation. The function of FAM149A is unknown. Recent genetic studies of deep vein thrombosis (DVT) reported that several common single nucleotide polymorphisms (SNPs) in the *F11*, *KLKB1*, and *CYP4V2* region (not including *FAM149A*) were associated with DVT and plasma FXI antigen levels (individuals with FXI antigen in 90 percentile having a 2-fold increased risk for DVT) [3]. The two SNPs that were independently associated with DVT and high plasma FXI antigen levels were located within *F11* gene (rs2036914 and rs2289252) [9]. SNP rs13146272 within *CYP4V2* was associated with DVT and FXI levels also after adjustment for rs2036914 and rs2289252 [9]. Of note, these three SNPs remain associated with DVT after adjustment for FXI levels. Possibly average levels are not so much affected by these SNPs, but rather that peak levels (e.g. as a result of hormone stimuli) are affected. It could also be that qualitative changes in the FXI protein not detected by quantitative effects are responsible (e.g. missense or splice changes). Alternatively, SNPs in *F11* and *CYP4V2* may also affect the expression of KLKB1, which is clearly also a strong candidate DVT risk gene. The above suggests that the DVT risk associated with variation in the 4q35.2 region may not necessarily be solely attributed to *F11* gene variation and/or FXI protein levels. Furthermore, it opens the possibility that *KLKB1* and *CYP4V2* may be more than just neighbors of *F11*, but may also share regulatory elements.

Experimental evidence for the possible regulatory interaction of the genes within 4q35.2 region should follow from a detailed *in vitro* analysis of this cluster, but is complicated as this requires expression analysis of the entire cluster with (the as yet unidentified) regulatory elements in their natural positions. Such analysis is limited by the size of the cluster (~100 kb). In mice, *F11*, *Klkb1* and *Cyp4v3* (the mouse orthologue of *CYP4V2*) are also located together (Figure 1). Furthermore, in mice, hepatic *F11* transcription, unlike many other coagulation proteases, is clearly responsive to changes in estrogen status [10], thyroid hormone status [unpublished observation] or dietary fat intake [11]. Here, we took advantage of mouse hepatic *F11* transcriptional responsiveness to obtain additional evidence for possible regulatory interaction of the genes within the 4q35.2 locus. To this end, we studied variation in hepatic *F11*, *Klkb1* and *Cyp4v3* transcript levels under a number of conditions that affect mouse hepatic *F11* gene transcription. We observed parallel responses in *F11* and *Cyp4v3* transcript levels when hepatocyte nuclear factor 4α (HNF4α), estrogen, and dietary status were varied. In contrast, *Klkb1* only showed an *F11*-like response for the fat feeding condition.

## Materials and Methods

### Materials

Livers from 45-day-old female liver-specific *Hnf4a*-null mice with a liver-specific deletion of exons 4 and 5 of the *Hnf4a* gene (*Hnf4a*-floxed/floxed with albumin-Cre; KO) or control mice (*Hnf4a*-floxed/floxed without albumin-Cre; FLOX) were described previously [12]. Liver from 10-week-old ovariectomized female C57BL/6J mice treated orally with synthetic estrogen hormone (17α-ethinylestradiol) (1µg) once for ‘single doses’ or daily for 10 days for ‘multiple doses’ and respective vehicle-treated controls have been described previously [10].

### Animal experiments

C57BL/6J males and females were purchased from Charles River Laboratories (Maastricht, the Netherlands) and housed under a 12-h light/dark cycle, with standard chow diet and drinking water provided ad libitum. For studying the impact of the natural estrogen 17ß-estradiol, 8-week-old female mice were bilaterally ovariectomized under isoflurane anesthesia, and after a 2 week recovery period, they were randomly assigned to either the experimental group or vehicle treatment group. 17ß-estradiol (Sigma Aldrich, Steinheim, Germany) stock was prepared in ethanol and diluted in arachid oil at a final concentration of 1% ethanol prior to injection. 17ß-estradiol was injected subcutaneously once or for 5 days, at a (daily) dose of 2 µg per mouse. For the vehicle treatment, mice were subcutaneously injected with 100 µL of arachid oil with an ethanol concentration of 1%.

For study the effect of thyroid hormone, male C57BL/6J mice, 8 weeks of age, were fed a low iodine diet (ICN Biomedicals, Inc., Aurora, OH) and drinking water supplemented with 1% (wt/vol) potassium perchlorate (Sigma). 3,3′,5-triiodo-L-thyronine sodium salt (T_3_) (Sigma Aldrich) stocks of 1 mg/ml were prepared in 4 mM sodium hydroxide and stored at 4 degrees Celsius. For injection, a T_3_ stock was diluted to 2.5 µg T_3_/ml in phosphate buffered saline supplemented with 0.02% bovine serum albumin with a final concentration of sodium hydroxide of 0.2 mM. Mice received a daily intraperitoneal injection of (0.5 µg) 200 µl thyroid hormone solution for 14 days.

For studying the effect of fat feeding, 8-week-old male C57BL/6J mice were fed a low-fat control diet (LFD; 10% energy in the form of fat, D12450B, Research Diet Services, The Netherlands) as a run-in for a period of 2 weeks. Subsequently, they were randomly assigned to either the experimental group fed a high-fat diet (HFD; 45% energy in the form of fat, D12451, Research Diet Services, The Netherlands) or maintained on the control LFD for 1 or 7 days.

For small interfering (si) RNA-mediated knockdown of HNF4α in 8-week-old female mice, a control siRNA (siNEG; cat. # 4404020, Ambion, Life Technologies Corporation, USA) and a siRNA tested in mouse primary hepatocytes to be effective to reduce HNF4α transcript and protein levels by 90% at a concentration of 3 nM were used as described previously [13]. The sequences of the two siHNF4α RNA-strands were, sense: 5 ´-AGA GGU CCA UGG UGU UUA AUU-3 ´ and antisense: 5 ´-UUA AAC ACC AUG GAC CUC UUG-3 ´ (siHNF4α, cat. # 67635). Control siNEG (catalogue number 4404020) and siHNF4α were complexed with Invivofectamine® 2.0 (Invitrogen, Life technologies Corporation, USA) exactly according to the manufacturer’s protocol. Subsequently, C57BL/6J mice were intravenously injected via the tail vein with 200µl complexed siRNA, a dose of 7 mg of siRNA per kg body weight.

At the indicated time points, 17ß-estradiol-treated, T_3_-treated, fat-fed, or siHNF4α injected animals and the respective controls were anesthetized by a subcutaneous injection with a mixture of ketamine (100 mg/kg), xylazine (12.5 mg/kg) and atropine (125 µg/kg) after which the abdomen was opened by a midline incision and a blood sample on sodium citrate (final concentration 0.32%) was drawn from the inferior caval vein. Plasma was obtained by centrifugation and stored at -80°C until use [10]. Liver was isolated and weighed, and part of a liver lobule was snap-frozen for mRNA analyses and stored at -80°C until use. All experimental procedures were approved by the animal welfare committee of the Leiden University (under registration # 10244, 10032 and 11005).

### Hepatic transcript and plasma protein analyses

RNA was isolated from mouse livers and subsequently analyzed for transcripts by quantitative real-time PCR as described previously [10]. The gene-specific quantitative primers used are presented in Table S1. β-actin was used as an internal control for normalization and quantification. The ΔC_t_ values of the individual samples were related to the mean ΔC_t_ of the reference group (i.e ΔΔC_t_). Values are expressed as mean (2 POWER of mean ΔΔC_t_) with a lower range (2 POWER of mean ΔΔC_t_+SEM) and an upper range (2 POWER of mean ΔΔC_t_-SEM).

Plasma FXI activity levels were determined as described previously [10]. Respective control groups were used as a reference.

### Statistical analyses

Data were analyzed with ‘GraphPad Instant’ software and statistical differences were assessed using the Student’s t-test (plasma analysis) or the Mann-Whitney Rank sum test (transcript levels). The Pearson correlation coefficient (r) was used to evaluate whether hepatic transcript levels were correlated. A *p*-value <0.05 was considered to be significant.

## Results

**Figure 2:**
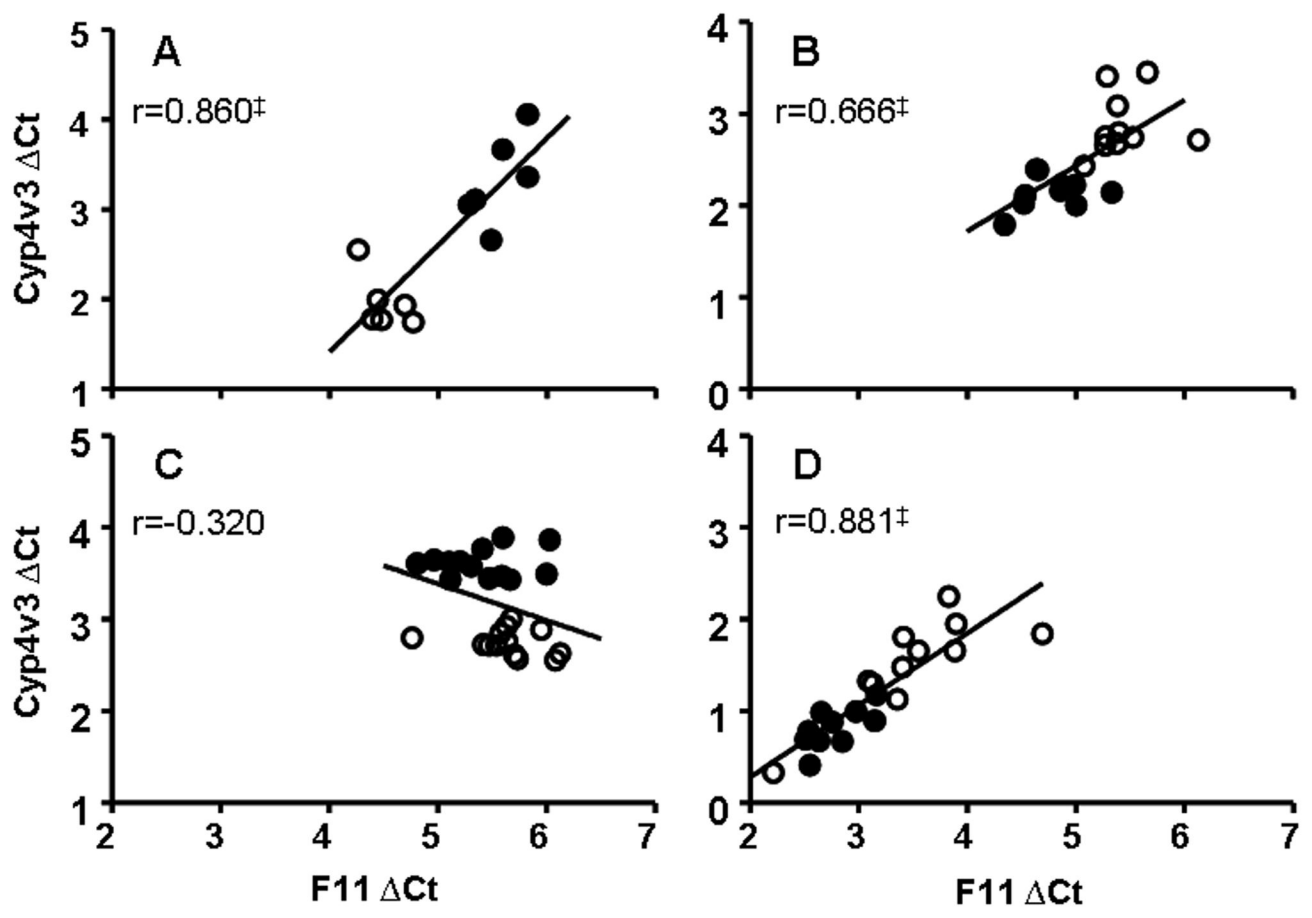
Correlation between *F11* hepatic transcript levels and *Cyp4v3* under different metabolic conditions. (A) siRNA-mediated HNF4α (●) depletion or control siRNA (○) in mouse liver, (B) 10 days of oral EE (●) or vehicle (○) treatment in ovariectomized mice, (C) 14 days of T_3_ (●) or vehicle treatment (○) and (D) mice fed with high (●) or low (○) fat diet for one day. Correlations were determined using Pearson correlation coefficient (r). *p*-values < 0.05 were regarded as statistically significant. ^‡^ *p* < 0.001.

To find support for the hypothesis of common regulatory elements in the 4q35.2 locus, we examined whether *F11*, *Klkb1* and *Cyp4v3* transcription in mice responds in a concerted manner to stimuli affecting *F11* transcript levels i.e. estrogen hormone [10] and thyroid hormone treatments (unpublished observation) and fat feeding [10,11]. Before doing so, we first investigated the regulatory role of hepatocyte nuclear factor 4α (HNF4α) for the genes located in this locus, as this transcription factor controls the *F11* gene [14]. In livers of 45-day-old mice lacking HNF4α in liver (KO), the *F11* transcript levels and *Cyp4v3* levels were strongly reduced or even absent when compared to their control littermates (FLOX) (-96 and -90%, respectively; Table 1). For *Klkb1*, located in between the *F11* and *Cyp4v3*, transcript levels were also clearly affected by HNF4α status, but to a lesser extent (-48%, Table 1). As these reducing effects may be secondary to changes in liver physiology due to prolonged (45 days) ablation of hepatic HNF4α, transcript levels of the gene cluster in adult C57BL/6J mice, in which hepatic *Hnf4a* was rapidly reduced by means of a liver-targeted specific small interfering (si) RNA were also examined. Two days after intravenous injection of a double-stranded siRNA known to be effective for *Hnf4a* knockdown, a 61% reduction of hepatic *Hnf4a* transcript levels was observed that correlated with the reduction in *F11* and *Cyp4v3* transcript levels (-52% and -61% respectively, Table 1). *Klkb1* transcript levels were not affected by the HNF4α siRNA. At reduced hepatic HNF4α RNA levels, *F11* and *Cyp4v3* transcript levels in the individual mice strongly and significantly correlated (Figure 2A; Table 2), whereas *F11* and *Klkb1* were not significantly correlated (Table 2). Furthermore, *Hnf4a* transcript levels significantly correlated with *F11* and *Cyp4v3*, but not with *Klkb1* (Figure S1). The specificity of the effects of HNF4α modulation (KO or in siRNA-mediated knockdown) on *F11*, *Cyp4v3* and to a lesser extent *Klkb1* transcription are illustrated by the absence of effects for hepatic transcript levels of coagulation factor genes *F2*, *F7*, and *F10*, while that of the established HNF4α target *F12* was clearly reduced (Table 3).

**Table 1:** Transcript levels of *F11*, *Klkb1* and *Cyp4v3* in livers of challenged mice.

	**n**	**F11**	**Klkb1**	**Cyp4v3**
**Hepatic HNF4α status**				
siNEG mice	6	1 (0.95-1.05)	1 (0.95-1.05)	1 (0.92-1.08)
siHNF4α mice	6	0.48 (0.45-0.52)^†^	0.92 (0.88-0.97)	0.39 (0.34-0.45)^†^
FLOX mice	8	1 (0.89-1.13)	1 (0.91-1.11)	1 (0.94-1.06)
KO mice	8	0.04 (0.04-0.05)^‡^	0.52 (0.50-0.54)^‡^	0.10 (0.09-0.11)^‡^
**Estrogen hormone**				
Vehicle control	5	1 (0.91-1.10)	1 (0.96-1.04)	1 (0.95-1.05)
EE (1µg, 5 hours)	5	1.35 (1.29-1.42)*	1.20 (1.14-1.27)*	1.23 (1.17-1.29)*
Vehicle control	10	1 (0.94-1.07)	1 (0.93-1.07)	1 (0.93-1.08)
EE (1µg/day, 10days)	10	1.59 (1.49-1.69)^‡^	1.10 (1.04-1.16)	1.65 (1.59-1.72)^‡^
Vehicle control	7	1 (0.95-1.05)	1 (0.93-1.07)	1 (0.93-1.08)
E2 (2µg/day, 24h)	7	1.31 (1.20-1.43)*	1.04 (0.98-1.11)	1.09 (1.04-1.14)
Vehicle control	7	1 (0.92-1.09)	1 (0.92-1.08)	1 (0.94-1.07)
E2 (2µg/day, 5days)	7	1.62 (1.46-1.80)^‡^	1.02 (0.97-1.08)	1.00 (0.96-1.05)
**Thyroid hormone**				
Vehicle control	13	1 (0.94-1.07)	1 (0.97-1.03)	1 (0.97-1.03)
T_3_ (0.5µg/day, 14days)	13	1.18 (1.10-1.26)*	0.92 (0.89-0.95)	0.55 (0.54-0.57)^‡^
**Feeding condition**				
Low fat control	11	1 (0.89-1.13)	1 (0.95-1.05)	1 (0.90-1.11)
High fat (1 day)	10	1.70 (1.61-1.76)^‡^	1.49 (1.44-1.53)^‡^	1.67 (1.60-1.76)^†^
High fat (7 days)	8	1.58 (1.42-1.76)^‡^	1.32 (1.25-1.38)^†^	1.73 (1.66-1.80)^†^

Data are expressed as 2 POWER of mean ΔΔCt with lower and upper range. β-actin was used as internal control for quantification and normalization. The ΔCt values of the individual samples were related to the mean ΔCt of the reference group. *p<0.05, ^†^ *p*<0.01, ^‡^ *p*<0.001.

siNEG/siHNF4α mice; mice injected with control (negative) or HNF4α siRNA respectively, KO/FLOX mice; HNF4α conditional liver knockout mice and control littermates, respectively, EE; ethinylestradiol, E2; 17-β estradiol, T_3_; 3,3′,5-Triiodo-L-thyronine.

**Table 2:** Correlation between transcript levels of *F11*, *Klkb1* and *Cyp4v3* in livers of challenged mice.

	***F11*-*Cyp4v3***		***F11*-*Klkb1***	
	***r***	***p*-values**	***r***	***p*-values**
**Hepatic Hnf4α status**				
siNEG/siHNF4α mice	0.860	0.0003	0.307	0.332
FLOX/KO mice	0.994	< 0.0001	0.886	< 0.0001
**Estrogen hormone**				
Vehicle/EE (1µg, 5hours)	0.703	0.0233	0.475	0.165
Vehicle/EE (1µg/day, 10days)	0.666	0.0001	0.277	0.2518
Vehicle/E2 (2µg, 24hours)	0.541	0.0457	0.640	0.014
Vehicle/E2 (2µg/day, 5days)	0.333	0.2450	0.254	0.4032
**Thyroid hormone (T3**)				
T_3_ (0.5µg/day, 14days)	-0.320	0.1110	0.281	0.164
**Feeding condition**				
High fat (1 day)	0.881	< 0.0001	0.716	0.0003
High fat (7 days)	0.778	< 0.0001	0.546	0.019

Correlation between hepatic *F11* transcript levels and *Cyp4v3* (*F11*-*Cyp4v3*) or *Klkb1* (*F11*-*Klkb1*) under different metabolic conditions. Data was statistically analyzed with Pearson correlation coefficient (r). *p*-values < 0.05 were regarded as statistically significant.

siNEG/siHNF4α mice; mice injected with control (negative) or HNF4α siRNA respectively, KO/FLOX mice; HNF4α conditional liver knockout mice and control littermates, respectively, EE; ethinylestradiol, E2; 17-β estradiol, T_3_; 3,3′,5-Triiodo-L-thyronine.

**Table 3:** Transcript levels of *F2, F7, F10* and *F12* in livers of challenged mice.

	**n**	**F2**	**F7**	**F10**	**F12**
**Hepatic HNF4α status**					
siNEG mice	6	1 (0.96-1.05)	1 (0.96-1.05)	1 (0.97-1.04)	1 (0.97-1.04)
siHNF4α mice	6	0.90 (0.88-0.93)	1.02 (0.97-1.06)	1.04 (0.96-1.13)	0.65 (0.58-0.72)^†^
FLOX mice	8	1 (0.93-1.08)	1 (0.89-1.13)	1 (0.90-1.11)	1 (0.92-1.09)
KO mice	8	1.17 (1.11-1.22)	0.97 (0.93-1.01)	0.84 (0.79-0.89)	0.05 (0.04-0.06)^‡^
**Estrogen hormone**					
Vehicle control	5	1 (0.90-1.11)	1 (0.96-1.05)	1 (0.89-1.13)	1 (0.94-1.07)
EE (1µg, 5 hours)	5	0.75 (0.72-0.78)*	0.75 (0.72-0.77)^‡^	0.75 (0.71-0.79)	0.72 (0.67-0.78)*
Vehicle control	10	1 (0.95-1.05)	1 (0.89-1.13)	1 (0.95-1.05)	1 (0.96-1.04)
EE (1µg/day, 10days)	10	0.62 (0.58-0.66)^‡^	0.55 (0.53-0.56)^‡^	0.52 (0.50-0.55)^‡^	0.76 (0.73-0.80)^†^
Vehicle control	7	1 (0.95-1.06)	1 (0.92-1.09)	1 (0.94-1.06)	1 (0.93-1.08)
E2 (2µg/day, 24h)	7	1.03 (0.98-1.08)	1.08 (1.04-1.12)	1.15 (1.07-1.23)	1.09 (1.04-1.15)
Vehicle control	7	1 (0.97-1.04)	1 (0.96-1.04)	1 (0.96-1.04)	1 (0.92-1.08)
E2 (2µg/day, 5days)	7	0.84 (0.79-0.89)^†^	0.83 (0.79-0.88)^†^	0.89 (0.84-0.94)	0.85 (0.81-0.90)
**Thyroid hormone**					
Vehicle control	13	1 (0.96-1.05)	1 (0.97-1.03)	1 (0.95-1.05)	1 (0.95-1.05)
T_3_ (0.5µg/day, 14days)	13	0.65 (0.61-0.68)^‡^	0.96 (0.90-1.02)	0.73 (0.70-0.77)^‡^	1.24 (1.17-1.31)^†^
**Feeding condition**					
Low fat control	12	1 (0.95-1.05)	1 (0.96-1.04)	1 (0.97-1.04)	1 (0.97-1.03)
High fat (1 day)	10	1.17 (1.11-1.23)*	1.13 (1.07-1.19)	1.11 (1.05-1.18)	1.13 (1.08-1.19)
High fat (7 days)	8	1.03 (1.00-1.06)	1.00 (0.97-1.03)	1.02 (0.99-1.04)	1.03 (0.98-1.08)

Data are expressed as 2 POWER of mean ΔΔCt with lower and upper range. β-actin was used as internal control for quantification and normalization. The ΔCt values of the individual samples were related to the mean ΔCt of the reference group. *p*-values < 0.05 were regarded as statistically significant. *p<0.05, ^†^ *p*<0.01, ^‡^ *p*<0.001.

siNEG/siHNF4α mice; mice injected with control (negative) or HNF4α siRNA respectively, KO/FLOX mice; HNF4α conditional liver knockout mice and control littermates, respectively, EE; ethinylestradiol, E2; 17-β estradiol, T_3_; 3,3′,5-Triiodo-L-thyronine.

Subsequently, the effect of estrogen on transcription in the locus harboring *F11*, *Klkb1* and *Cyp4v3* was determined. In mice, estrogens have an overall downregulatory effect on liver coagulation gene transcription, but upregulated *F11*. Both oral administration of the synthetic estrogen 17α-ethinylestradiol (EE) for 10 days (1 µg/mouse/day) and subcutaneous injection of the natural estrogen 17ß-estradiol (E2) for 5 days (2 µg/mouse/day) to ovariectomized C57BL/6J mice induced moderate reductions of hepatic transcript for coagulation factor genes F2, F7, F10 and F12 as compared to the respective vehicle control treated animals (-38, -45, -48 and -24% for EE and -16, -17, -11 and -15% for E2, respectively, Table 3). However, the reduction in *F10* and *F12* transcript levels under E2 did not reach statistical significance. Although more subtle, for EE, but not for E2, these reducing effects were already apparent at 5 hours after a single dose of hormone. As expected [10], the opposite was observed for *F11*, with significant increased hepatic transcript levels upon prolonged exposure to both EE and E2 (+59% and +62%, respectively; Table 1). These changes were apparent at 5 hours after a single dose of EE (+35%) and E2 (+31%) (Table 1). For EE, but not for E2, the transcriptional response of *Cyp4v3*, but not that of *Klkb1*, largely resembled the response of *F11*. Consequently, also under EE significant correlations were only found for *F11* and *Cyp4v3* transcript levels. Although *Klkb1* and *Cyp4v3* were not affected by E2, we found significant correlations with *F11* at 24 hours (Figure 2B, Table 2).

Next, the modulation of the *F11*, *Klkb1* and *Cyp4v3* harboring locus by thyroid hormone was determined. C57BL/6J mice with suppressed endogenous thyroid hormone production were treated with triiodothyroxine (T_3_; 0.5 µg T_3_ per mouse for 14 days). T_3_ results in a decrease in most hepatic coagulation gene transcript levels, with downregulatory effects of -35 and -27% observed for *F2* and *F10* respectively, while *F7* levels remained unchanged (Table 3). In contrast, *F11 and F12* displayed a statistically significant increase in transcription (+18 and +24% as compared to vehicle, Tables 1 and 3). These upregulatory effects required sustained elevation of T_3_ as none of the transcripts analyzed demonstrated an immediate response at 4 hours after a single injection of T_3_ (data not shown). Increased hepatic *F11* transcript levels at 14 days of T_3_ treatment coincided with a 45% decrease in *Cyp4v3* transcript levels, while *Klkb1* levels were not significantly affected. Consequently, under changing in T_3_ levels, significant correlations between hepatic *F11*, *Klkb1* and *Cyp4v3* transcript levels were not observed (Figure 2C, Table 2).

High fat feeding of mice also affects transcription of *F11* [11]. Thus transcription of our genes of interest was assessed in livers of C57BL/6J mice that were fed a low-fat diet for 2 weeks (10 kcal% fat; LFD) as a run-in followed by a switch to a high-fat diet (45 kcal% fat; HFD) known to induce obesity when administered long-term (16 weeks [11]). Controls were maintained on the LFD control diet. Short exposure to the fat-rich diet (1 or 7 days) strongly induced *F11* transcript levels (+70 and +58% for 1 or 7 days, respectively), without significantly altering the hepatic transcript levels of *F2* (except at 1 day HFD), *F7*, *F10* or *F12* (Table 3). For both time points, increased *F11* transcript levels coincided with increased *Cyp4v3* and *Klkb1* transcript levels with strong correlations (Figure 2D, Table 2).

**Figure 3:**
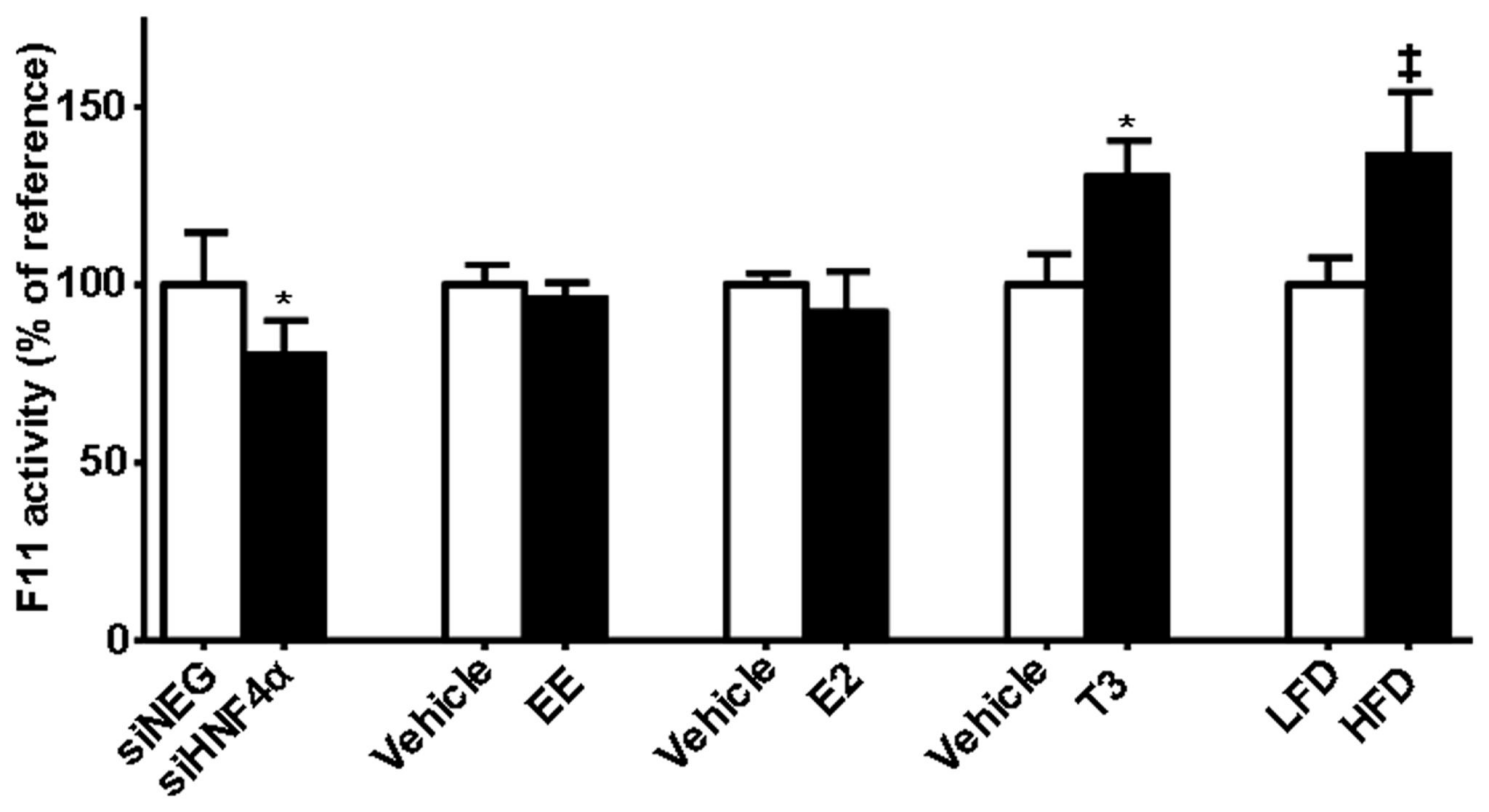
Plasma FXI activity under several metabolic conditions. Plasma FXI activity was measured under different metabolic conditions i.e siRNA-mediated depletion of HNF4α (siHNF4α) / siNEG (control siRNA), ovariectomized mice treated with vehicle / EE for 10 days or with E2 for 5 days, mice treated with vehicle / T_3_ for 14 days or mice were fed with low (control) / high fat diet for 1 day. Same mice were used for plasma and liver transcript analysis. For number of animals ‘n’ see Table 1. Data are represented as percentage of reference group ± standard deviation; data were statistically analyzed using the Student’s t-test. *p*-values < 0.05 were regarded as statistically significant. *p < 0.05, ^‡^ *p* < 0.001 vs reference group.

The above study conditions affecting mouse hepatic *F11* (*Klkb1* and *Cyp4v3*) transcription also affected the mouse FXI protein activity at the level of the plasma. This resulted in statistically significant correlations between hepatic *F11* transcript levels and plasma FXI activity, with the exception of synthetic and natural estrogen treatments (Figure 3, Table S2).

## Discussion

In the present study, we searched for evidence for the existence of common regulatory elements within a locus that has been linked to deep vein thrombosis and harbors *F11, KLKB1* and *CYP4V2* i.e. the 4q35.2 locus. The size of this gene cluster hampers *in vitro* analysis of the interaction of the genes within the 4q35.2 region. Therefore as an alternative, we used mice to study the regulation of the orthologous genes in mice under conditions that modulate transcription of the *F11* gene. Upon modulating hepatic HNF4α levels, estrogen status, thyroid hormone status or dietary fat intake, a significant effect on hepatic *F11* transcription was observed in a setting where transcription of other coagulation proteases was not, or differentially, affected. In contrast, parallel responses in *F11* and *Cyp4v3* transcript levels were produced when HNF4α, estrogen, and dietary status were varied. *Klkb1* showed an *F11*-like response for the fat feeding condition only. Thus, we conclude that in mice, hepatic *F11* and *Cyp4v3* display parallel transcriptional responses suggesting the presence of shared regulatory elements. Possible concerted regulation does not include the *Klkb1* gene, despite the fact that it is located in between these two genes.

Co-regulation of the transcription of genes in close proximity of one another, as the *F11* and *Cyp4v3* genes, may be the result of two different mechanisms that are not mutually exclusive. On one extreme, the genes may be under the control of a common enhancer that stimulates transcription of those nearby promoters with which it can form productive transcription factor complexes. On the other end of the spectrum, co-regulation may be the result of the presence of binding sites of comparable importance and activity for the same transcription factor in the various promoters. A combination of both scenarios is also possible. In the case of a common enhancer, the genes need to be in close proximity of one another, in case of the presence of similar transcription factor binding sites in their promoters, their relative position is not necessarily important; the genes might have been on different chromosomes and the same effect would have been observed. The present mouse study shows that sufficient HNF4α levels are crucial to the regulation of *F11* and *Cyp4v3* (Figure S1, Table 1). This suggests that HNF4α, at ~50% levels of normal, is the limiting factor in the transcription complexes regulating the expression of *F11* and *Cyp4v3*. On the basis of these data, one would predict that HNF4α regulatory element(s) are present near the murine promoters and/or enhancers of the *F11* and *Cyp4v3* genes. Indeed, the mouse and human *F11* loci are predicted to carry multiple functional HNF4α binding sites [15,16]. Hence, such HNF4α binding sites are candidate for the common regulatory sequence within 4q35.2 locus.

Using Genomatix MatInspector and JASPAR programs, we identified two putative HNF4α binding sites in the enhancer (~5kb upstream of the start-site) and one in the promoter region (located just upstream of the transcription start site) of *F11* gene. No binding sites for estrogen receptor α and thyroid hormone receptor were identified using these softwares. In addition, direct comparison of the human and mouse (enhancer and promoter) sequences did not show very convincing conservation of the putative binding sites. This exemplifies that a reliable identification of functional binding sites requires, perhaps unsurprisingly, functional data. These were beyond the scope of the present manuscript.

It was proposed that the human SNPs associated with thrombotic risk may modulate FXI expression in response to changing age and hormonal levels [9]. Our mouse data demonstrate that the cluster harboring *F11*, *Klkb1* and *Cyp4v3* is highly responsive to changes in hormones and metabolism. Of note, these changes are much larger than, and in a different direction from the alterations in the expression of coagulation genes *F2*, *F9*, *F10*, *F12*, which were included as controls in the analysis. Both single/multiple estrogen doses were able to modulate *F11* hepatic transcription. Studies indicated that there are ERα binding sites near the *F11* gene [17] which explains immediate transcriptional response of *F11*. Alternatively, prolonged thyroid hormone exposure was required to evoke a clear *F11* transcriptional response. This could be explained by an indirect modulation involving an intermediate transcription factor (e.g. HNF4α) additional to thyroid hormone receptor. A small number of studies reported that estrogens, thyroid hormone and dietary fat may affect hepatic HNF4α [18-20]. Although hepatic HNF4α transcript and protein levels were not affected (both) in the conditions mentioned above (Figure S2), this does not exclude that HNF4α transcriptional activity is affected by hormones and diet thereby contributing to the observed possible concerted regulation of *F11* and *Cyp4v3*. Alternatively, dietary fat can induce acute hepatic stress and inflammation [11,21] that involving many transcription factors. FOXA1 is one of such transcription factor [22] and ChIP-seq data from the ENCODE consortium [23] indicates that there are FOXA1 binding sites near the *F11* gene. Whether stress-related FOXA1 or HNF4α contribute to possible concerted regulation in the human 4q35, 2 locus has not been studied.

It was also suggested that human SNPs in *F11* and *CYP4V2* may impact the expression of KLKB1, and thereby increase risk for DVT [18-20]. The mouse data do not provide additional support for regulatory interaction between *F11* and *CYP4V2* on one hand and KLKB1 on the other hand, and make modulation of DVT risk through impact on *KLKB1* by SNPs in *F11* and *CYP4V2* less likely.

The mouse data encouraged us to investigate whether SNP rs2036914, rs2289252 and rs13146272 within the 4q35.2 locus are related to (common) regulatory sequences for this locus. SNPs rs2036914 and rs2289252 are located in introns of the *F11* gene and ChIP-seq analysis for HNF4α or FOXA1 binding sites, among others, does not provide any evidence for transcription factor binding sites in these regions. Also, the sequences around the SNPs are not strongly conserved in mammals. rs13146272 is a missense mutation in *CYP4V2* and this position is relatively well conserved in other species and in other CYPs. It might be speculated that this SNP alters sensitivity or specificity of *CYP4V2* and thereby affects plasma lipid levels, as some mutations in *CYP4V2* have been shown to do [24]. Thus, SNP rs2036914, rs2289252 and rs13146272 do not seem to be present in regulatory sequences in the 4q35.2 locus. However, it should be stressed that for all three SNPs, it is still entirely possible that they are merely genetically linked to other causal/functional SNPs which do impact regulation of the locus.

Parallel responses in *F11* and *Cyp4v3* transcript levels were produced when HNF4α and dietary status were varied. Such responses were also produced upon variation in estrogen, however minimal or not when using 17ß-estradiol (E2) (Tables 1 and 2). Overall ethinylestradiol (EE) treatment had stronger impact on hepatic transcription as compared to 17ß-estradiol (compare also the effects of the two compounds on *F2*, *F7*, *F10* and *F12* transcript levels). In contrast to 17ß-estradiol, ethinylestradiol is orally effective and thereby likely results in more effective exposure of the liver to the estrogenic compound as compared to a subcutaneous administration (17ß-estradiol). This is in line with our earlier observations using these compounds [10]. For thyroid hormone *F11* and *Cyp4v3* transcript levels were affected in opposite direction. We have no explanation for these opposite effects, but these observations highlight that, although mouse *F11* and *Cyp4v3* may share regulatory elements, the transcription of these two genes is clearly not under control of one single shared regulatory region.

Remarkably, only for the estrogen treatment conditions, changes in *F11* transcript levels did not translate into increased FXI plasma protein levels, at least at the level of protein activity (Table 1, Figure 3). This phenomenon was observed in multiple experiments [10]. Whether estrogen possibly induces increased plasma FXI antigen, but not FXI protein activity, or whether estrogen increases FXI protein clearance and degradation thereby masking the effects on transcription on protein levels, has not been studied. Alternatively, estrogen may affect other (unknown) mechanism that influence FXI activity such as the post-translational modifications. Thus, whether changes in hepatic *F11* transcript levels truly were not translated into changes in plasma FXI (protein and activity) is at present subject to speculation. Although not determined, it would also be interesting to determine whether effects on hepatic *Cyp4v3* transcription translate to effects on hepatic Cyp4v3 protein (activity) level. This would shine a light on whether parallel transcriptional responses of *F11* and *Cyp4v3* extends to protein level.

In conclusion, our mouse data demonstrate that within the mouse *F11*, *Klkb1*, *Cyp4v3* gene cluster, in particular *F11* and *Cyp4v3*, frequently display a striking parallel transcriptional response suggesting the presence of regulatory elements. We speculate that, if present, SNPs within the human orthologues of these unidentified elements could causally influence DVT risk.

## Supporting Information

Figure S1 **Correlation between hepatic *Hnf4a* and *F11* (A), *Hnf4a* and *Klkb1* (B) and *Hnf4a* and *Cyp4v3* (C) under siRNA-mediated HNF4α knockdown (●) and control siRNA (○) in mouse liver.** Data were statistically analyzed with Pearson correlation coefficient (r). *p*-values <0.05 were regarded as statistically significant. ‡*p* < 0.001.
(TIF)

Figure S2 **Hepatic transcript and protein levels of HNF4α under metabolically challenged conditions in mice.** (A) Hepatic transcript levels were determined by quantitative real-time PCR. Data are expressed as mean with error bars representing the difference between 2 POWER of upper and lower range of the mean ΔΔCt. β-actin was used as internal control for quantification and normalization. The ΔCt values of the individual samples were related to the mean ΔCt of the reference group. On the x-axis the metabolic conditions are depicted. Data were statistically analyzed using Mann Whitney Rank Sum Test. *P*-values less than 0.05 were regarded as statistically significant. *p<0.05. (B) Immunoblotting for HNF4α was perfromed for liver homogenates that were prepared for three randomly selected mice per condition. 15µg total protein lysate was loaded in each lane and HNF4α was detected using anti-HNF4α antibody (C-19, sc-6556, Santa Cruz Biotech, Santa Cruz, CA, USA). β-actin was used as a protein loading control.
siNEG/siHNF4α mice; mice injected with control (negative) or HNF4α siRNA respectively, EE; ethinylestradiol, E2; 17-β estradiol, T_3_; 3,3′,5-Triiodo-L-thyronine.
(TIF)

Table S1 (DOC)

Table S2 (DOC)
