# Supplementary figures and images for: Regulation of the F11, Klkb1, Cyp4v3 Gene Cluster in Livers of Metabolically Challenged Mice
Source: PLoS One. 2013 Sep 16;8(9):e74637. doi: 10.1371/journal.pone.0074637 (PMC3774739; doi:10.1371/journal.pone.0074637)

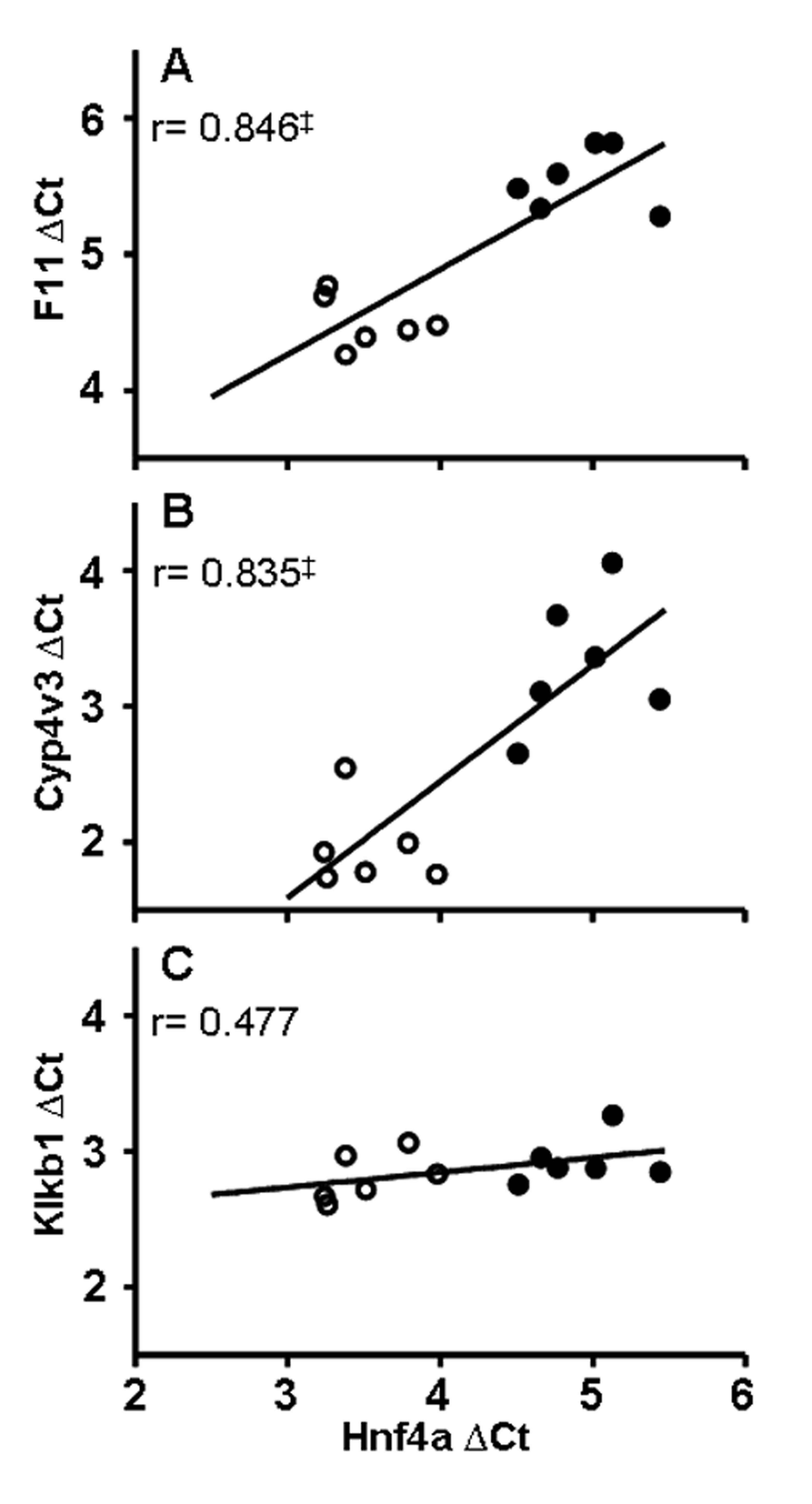

Supplement: Figure S1 — Correlation between hepatic Hnf4a and F11 (A), Hnf4a and Klkb1 (B) and Hnf4a and Cyp4v3 (C) under siRNA-mediated HNF4α knockdown (●) and control siRNA (○) in mouse liver. Data were statistically analyzed with Pearson correlation coefficient (r). p-values <0.05 were regarded as statistically significant. ‡p < 0.001. (TIF) [file pone.0074637.s001.tif]

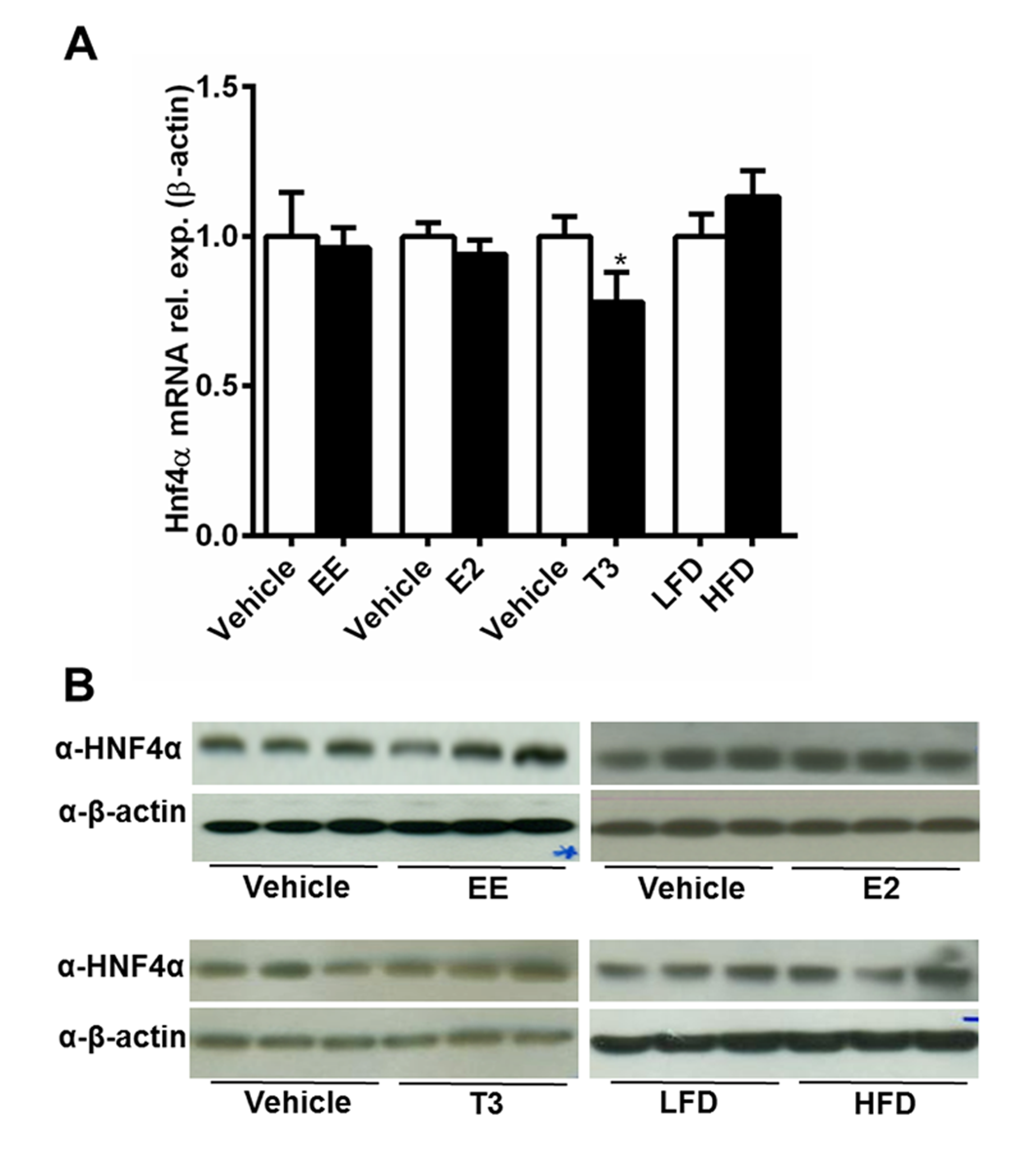

Supplement: Figure S2 — Hepatic transcript and protein levels of HNF4α under metabolically challenged conditions in mice. (A) Hepatic transcript levels were determined by quantitative real-time PCR. Data are expressed as mean with error bars representing the difference between 2 POWER of upper and lower range of the mean ΔΔCt. β-actin was used as internal control for quantification and normalization. The ΔCt values of the individual samples were related to the mean ΔCt of the reference group. On the x-axis the metabolic conditions are depicted. Data were statistically analyzed using Mann Whitney Rank Sum Test. P-values less than 0.05 were regarded as statistically significant. *p<0.05. (B) Immunoblotting for HNF4α was perfromed for liver homogenates that were prepared for three randomly selected mice per condition. 15µg total protein lysate was loaded in each lane and HNF4α was detected using anti-HNF4α antibody (C-19, sc-6556, Santa Cruz Biotech, Santa Cruz, CA, USA). β-actin was used as a protein loading control. siNEG/siHNF4α mice; mice injected with control (negative) or HNF4α siRNA respectively, EE; ethinylestradiol, E2; 17-β estradiol, T3; 3,3′,5-Triiodo-L-thyronine. (TIF) [file pone.0074637.s002.tif]
