# Supplementary material for: Regulation of the F11, Klkb1, Cyp4v3 Gene Cluster in Livers of Metabolically Challenged Mice
Source: PLoS One. 2013 Sep 16;8(9):e74637. doi: 10.1371/journal.pone.0074637 (PMC3774739; doi:10.1371/journal.pone.0074637)
Supplement: Table S1 — (DOC) [file pone.0074637.s003.doc]

**Table** S1: Quantitative real-time PCR primer sequences

| **Gene** | **Forward primer (5 ́-3 ́ )** | **Reverse primer (3 ́-5 ́)** |
| --- | --- | --- |
| *ß-actin* | AGGTCATCACTATTGGCAACGA | CCAAGAAGGAAGGCTGGAAAA |
| *F11* | GAAGGATACGTGCAAGGGAGATT | CAAGTGCCAGACCCCATTGT |
| *KLKB1* | TGGTCGCCAATGGGTACTG | ATATACGCCACACATCTGGATAGG |
| *CYP4V3* | CTCTCCGAGTTTTCCCATCTGT | TTGTAACCGCCCACTTCACA |
| *HNF4α* | AGAGGTTCTGTCCCAGCAGATC | CGTCTGTGATGTTGGCAATC |
| *F2* | GGACGCTGAGAAGGGTATCG | CCCCACACAGCAGCTCTTG |
| *F7* | CGTCTGCTTCTGCCTCCTAGA | ATTTGCACAGATCAGCTGCTCAT |
| *F10* | GCAAAACCGGGTCAAATCC | ACCTCCACAGAATGCCTCAATT |
| *F12* | GGGCTTCTCCTCCATCACCTA | GCAACTGTTTTTGCTTTCC |
