# Supplementary material for: Regulation of the F11, Klkb1, Cyp4v3 Gene Cluster in Livers of Metabolically Challenged Mice
Source: PLoS One. 2013 Sep 16;8(9):e74637. doi: 10.1371/journal.pone.0074637 (PMC3774739; doi:10.1371/journal.pone.0074637)
Supplement: Table S2 — (DOC) [file pone.0074637.s004.doc]

**Table S2: Correlation between hepatic transcript levels of *F11* and plasma FXI activity in challenged mice.**

|  | ***F11 transcript* – plasma FXI activity** | |
| --- | --- | --- |
|  | ***r*** | ***p*-values** |
| **Hepatic Hnf4α status** |  |  |
| siNEG/siHNF4α mice | 0.6787 | 0.0152 |
|  |  |  |
| **Estrogen hormone** |  |  |
| Vehicle/EE (1µg/day, 10days) | 0.0835 | 0.7363 |
|  |  |  |
| Vehicle/E2 (2µg/day, 5days) | 0.0796 | 0.7867 |
|  |  |  |
| **Thyroid hormone (T3)** |  |  |
| T_3_ (0.5µg/day, 14days) | 0.4399 | 0.0278 |
|  |  |  |
| **Feeding condition** |  |  |
| High fat (7 days) | 0.4734 | 0.0406 |

**Table S2:** Correlation between hepatic *F11* transcript levels and plasma FXI activity in respective mice under different metabolic conditions. Data was statistically analyzed with Pearson correlation coefficient (r). *p*-values < 0.05 were regarded as statistically significant.

siNEG/siHNF4α mice; mice injected with control (negative) or HNF4α siRNA respectively, EE; ethinylestradiol, E2; 17-ß estradiol, T_3_; 3,3′,5-Triiodo-L-thyronine.
